# Time-of-flight spectroscopy for laser-driven proton beam monitoring

**DOI:** 10.1038/s41598-022-25120-6

**Published:** 2022-12-12

**Authors:** Marvin Reimold, Stefan Assenbaum, Constantin Bernert, Elke Beyreuther, Florian-Emanuel Brack, Leonhard Karsch, Stephan D. Kraft, Florian Kroll, Markus Loeser, Alexej Nossula, Jörg Pawelke, Thomas Püschel, Hans-Peter Schlenvoigt, Ulrich Schramm, Marvin E. P. Umlandt, Karl Zeil, Tim Ziegler, Josefine Metzkes-Ng

**Affiliations:** 1 Helmholtz-Zentrum Dresden, Rossendorf, 01328 Dresden Germany; 2 Technische Universität Dresden, 01062 Dresden, Germany; 3 OncoRay – National Center for Radiation Research in Oncology, 01309 Dresden, Germany; 4 Martin-Luther-Universität Halle-Wittenberg, 06120 Halle, Germany

**Keywords:** Plasma-based accelerators, Laser-produced plasmas

## Abstract

Application experiments with laser plasma-based accelerators (LPA) for protons have to cope with the inherent fluctuations of the proton source. This creates a demand for non-destructive and online spectral characterization of the proton pulses, which are for application experiments mostly spectrally filtered and transported by a beamline. Here, we present a scintillator-based time-of-flight (ToF) beam monitoring system (BMS) for the recording of single-pulse proton energy spectra. The setup’s capabilities are showcased by characterizing the spectral stability for the transport of LPA protons for two beamline application cases. For the two beamline settings monitored, data of 122 and 144 proton pulses collected over multiple days were evaluated, respectively. A relative energy uncertainty of 5.5% (1$\upsigma$) is reached for the ToF BMS, allowing for a Monte-Carlo based prediction of depth dose distributions, also used for the calibration of the device. Finally, online spectral monitoring combined with the prediction of the corresponding depth dose distribution in the irradiated samples is demonstrated to enhance applicability of plasma sources in dose-critical scenarios.

Laser plasma-based accelerators (LPAs) for protons exploit the $\sim$ TV/m electric fields supported in plasmas to generate spectrally broadband particle pulses in the multi-10 MeV energy range on micrometer acceleration scales^1–3^. Owing to their inherent sub-ps duration at the source and high particle number per pulse ($\sim {10^{12}}$), LPA proton pulses are a unique tool for multiple applications including ultra-fast and broadband pumping and probing of materials or electromagnetic fields in plasma^4,5^. Moreover, the ultra-short and intense pulses provide pulse dose rates exceeding $10^{9}\hbox { Gy/s}$, a regime unreached at most conventional accelerators. Proton LPAs can hence complement the broad variety of radiation sources applied for ultra-high dose rate radiobiology^6^.

A wider dissemination of these application fields requires diagnostic tools capable of reliably characterizing LPA proton pulses which feature a large energy-dependent divergence of 200–400$\hbox { mrad}$ half opening angle^7,8^ and an exponentially decaying energy spectrum up to a maximum energy cut-off. Additionally, inherent pulse-to-pulse fluctuations of the source affecting angular and spectral shape as well as pulse intensity call for online single pulse characterization. Challenges for detection further arise from the harsh plasma environment in which proton LPA pulses are generated, featuring strong electromagnetic pulses (EMP)^9^, a mixed background radiation field of multiple ion species, electrons, X-rays and neutrons together with strong optical radiation from the laser pulse itself and emitted by the hot plasma.

These conditions have resulted in a broad detection method repertoire for LPA proton pulses. For state-of-the-art pulse characterization, experimental setups generally incorporate a combination of complementary devices featuring various detection principles, online and offline analysis and acceptance angles. These detectors include radiochromic films (RCFs)^10,11^, scintillators^12–15^ and ultra-sound-based detectors^16^ for dose-based measurements as well as Thomson parabola spectrometers (TPS)^11,17–19^ and time-of-flight (ToF) detectors^20–22^ for direct spectral characterization.

Moreover, application experiments call for dedicated detector systems to not only characterize the proton beam directly at the source but also also at the application site, normally located at a m-scale distance from the source. For applications in radiobiology, these detector systems include LPA-specific beam monitoring systems (BMS) and dosimetry setups^23,24^ which have been applied successfully for *in-vitro* radiobiological studies with 2D samples^25–28^. Recently, the increase in achievable LPA proton energies and numbers has enabled the transition from 2D (cell monolayer) to volumetric (e.g. tumor irradiation) mm- to cm-scale irradiation scenarios, e.g., for *in-vivo* radiobiology^29–31^. For dosimetric purposes, a volumetric dose distribution is generally decomposed in a relative lateral and depth dose distribution and an absolute dose at a reference point. To shape the required volumetric dose distributions from LPA proton pulses, several beamline concepts are in development and/or operation^29–36^. Here, the broad spectral distribution of the LPA proton source, in combination with its limited repetition rate, favors beamlines which allow selection and transport of ideally all spectral components required for the generation of a specific volumetric dose distribution. In this case, dose coverage of the volume intended for irradiation is achieved by a single ultra-short proton pulse and the ultra-high dose rate of the source is preserved for the irradiation. This efficient approach however comes at the prize of directly translating the LPA-inherent spectral fluctuations into fluctuations of the depth dose distribution in a sample. BMSs for volumetric irradiations consequently need to provide spectral information of the selected and transported proton pulse non-destructively with single pulse detection in order to complement the commonly monitored parameters like pulse intensity via ionization chambers (IC)^23^ or integrating charge transformers (ICT)^24,37^.

The unique combination of temporal and spectral structure of LPA proton pulses makes time-of-flight (ToF) spectrometry an ideal candidate for monitoring of volumetric irradiations: Spectrally filtered and transported proton pulses from state-of-the-art LPA beamlines typically feature $\sim 10\hbox { MeV}$ spectral bandwidth. The initial sub-ps pulse structure at the source is stretched to $\sim 10\hbox { ns}$ pulse duration within meter-scale flight distances by flight time broadening of the polyenergetic pulse. Moreover, the inherent synchronization of laser and proton pulses provides an easily accessible starting signal for the ToF measurement.

In this paper, we present a ToF spectrometer based on a plastic scintillator with nanosecond temporal resolution and its application as a BMS for spectrally filtered LPA proton pulses. Compatibility with the harsh plasma environment and particularly the EMP is ensured via optical scintillator readout and spatial separation between scintillator and the electronic components of the ToF spectrometer. The integration of the ToF BMS in an LPA proton beamline setup serves as testbed for the detector’s applicability in analyzing pulse-to-pulse fluctuations over a wide range of proton fluences. Beyond spectral characterization, core of the ToF BMS method is the deconvolution of the ToF signal from the detector response and consideration of all setup-specific signal corrections (e.g. flight time corrections due to energy loss along flight path) to enable a percent-level precise spectrum-based forward-calculation of the corresponding dose distribution via Monte-Carlo (MC) simulations. This allows for an *in-situ* approach for the ToF BMS device calibration by combining depth dose predictions with dose distribution measurements from RCFs. With these features, the presented ToF BMS is a novel approach not only to the critical task of spectral monitoring and tuning at proton LPA beamlines, but will also find its applications as a device for online spectral proton LPA source characterization.

**Figure 1:**
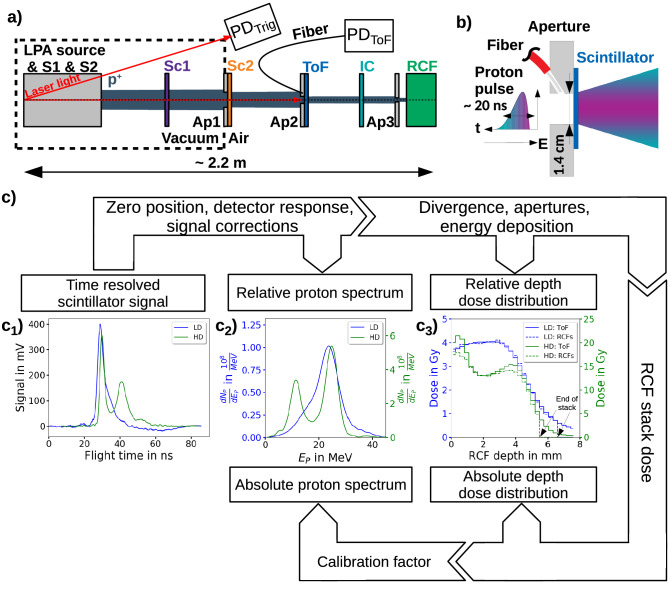
The setup and the operation of the time-of-flight (ToF) beam monitoring system (BMS) installed at the ALBUS-2S beamline for monitoring the transported kinetic proton energy spectrum of two different beamline settings (low dose per pulse (LD, blue lines) and high dose per pulse (HD, green lines)). (**a**) The ALBUS-2S beamline consisting of the laser plasma-based accelerator (LPA) source, two pulsed solenoids (S1 and S2) for proton beam transport and energy selection, several scatterers (Sc1 and Sc2) and apertures (Ap1, Ap2,Ap3) for beam homogenization and shaping^29^, the ToF BMS, the ionization chamber (IC) and radiochromic films (RCFs) for beam monitoring and dosimetry. The photodiode $\textrm{PD}_{\textrm{Trig}}$ is used for triggering the ToF BMS and the photodiode $\textrm{PD}_{\textrm{ToF}}$ for the ToF measurement. (**b**) The $20\hbox { ns}$-long proton pulse arrives at the ToF scintillator position with a lateral diameter of $30\hbox { mm}$ with the most energetic protons at the leading pulse edge due to flight time broadening. The proton divergence after the ToF aperture (illustrated with the colored cone) decreases with increasing kinetic proton energy, resulting from chromatic focusing of the solenoids and the kinetic energy dependent scattering behavior of the protons. (**c**) Workflow for obtaining the calibrated kinetic proton energy spectrum. Here, the measured time resolved $\textrm{PD}_{\textrm{ToF}}$ signal is used to calculate the proton flight time signal shown in $\textrm{c}_{1}$) and the relative kinetic proton energy spectrum. The forward simulation of the relative kinetic proton energy spectrum is used to predict the relative depth dose distribution in the RCF stack at the irradiation site. Comparison with the depth dose distribution measured with a calibrated RCF stack ($\textrm{c}_{3}$)) yields a calibration factor for the ToF BMS for obtaining the calibrated kinetic proton energy spectrum shown in $\textrm{c}_{2}$).

## Experimental setup

### ALBUS-2S beamline

The ToF BMS presented in this work is integrated into the LPA proton beamline ALBUS-2S^29^ installed at the Draco PW laser system^38,39^ at Helmholtz-Zentrum Dresden-Rossendorf (HZDR). The 2.2 m long beamline (distance from LPA proton source to irradiation site, Fig. 1a) is optimized to generate homogeneous mm- to cm-scale volumetric spatial dose distributions at an in-air irradiation site. Hence, ALBUS-2S allows to perform radiobiological studies^31^ with small animals and is also readily applicable for e.g., research and development of novel proton LPA detector systems^16^. In this setup the LPA proton source is powered by the Draco PW laser focused onto a $\sim 250\hbox { nm}$ thin plastic foil target. The Draco PW acts as the optical driver for the target normal sheath acceleration (TNSA) process, providing spectrally broadband proton pulses with cut-off energies above 70 MeV. For the transport of protons from the LPA source to the irradiation site, two pulsed solenoid magnets are used. The chromatic focusing properties of the pulsed solenoid magnets allow to actively select up to two proton energy bands from the source spectrum for transport by adjusting their respective magnetic field strength. Further passive spectral filtering with apertures and scattering foils with a $100{\upmu }\hbox { m}$-scale thickness made out of nickel and lead (see “Flight time reference, proton energy calculation and beam divergence” section) finetunes the transported spectrum. By adjusting laser pulse energy and hence proton source spectrum, the magnetic field strength of the solenoid and/or the apertures and scatter foils, application-adapted spatial dose distributions with a pulse dose range from $\sim 500\hbox { mGy}$ to multi-$10\hbox { Gy}$ can be generated at the irradiation site.

The general performance and capabilities of the ToF BMS will be showcased based on two different operation modes (low dose per pulse (LD)/ high dose per pulse (HD) mode) of the ALBUS-2S beamline, each optimized for specific radiobiological studies^31^. Both settings provide homogeneous dose distributions over a cylindrical volume with a diameter of $5\hbox { mm}$ and a depth of 4 mm and 3 mm, respectively. In the LD mode, a targeted total dose of 4 Gy is achieved via dose accumulation from a series of low dose pulses (from $330\hbox { mGy/pulse}$ to $800\hbox { mGy/pulse}$). This is necessary in order to reach the target dose with a deviation of $\le \pm 10\,{\%}$ and a depth dose inhomogeneity of $\le 10 {\%}$ over the target volume with 4 mm penetration depth (maximal dose difference as percentage of the applied mean dose value). In the HD mode, the single pulse dose of ALBUS-2S is escalated to dose levels of $> 10\hbox { Gy/pulse}$, maintaining the requirement of a depth dose inhomogeneity over 3 mm of $\le 10 {\%}$.

RCF stacks are irradiated for spatially resolved dose characterization in order to adjust beamline parameters to produce a dose distribution matching application requirements like dose value and homogeneity at the irradiation site. RCFs, however, are destructive offline detectors and require a scanning after irradiation to yield dose values. Fluctuations in the dose distribution caused by LPA source fluctuations are hence not readily resolved this way.

For ALBUS-2S, beam monitoring, i.e. online single proton pulse characterization, is available via a transmission-optimized IC, a method unable to distinguish between proton pulse intensity and spectral shape changes, which is insufficient in context of an LPA proton beamline like ALBUS-2S. The solution to these challenges is the direct spectral characterization of the transported proton pulse via a transmission ToF spectrometer as a BMS downstream of the spectral pulse shaping components.

### ToF BMS

The ToF BMS (Fig. 1b) presented in this work consists at its core part of a $200{\upmu }\hbox { m}$ thick, fast plastic scintillator slab with a temporal resolution of $\sim 0.55\hbox { ns}$ (1$\upsigma$) and a main emission wavelength of $370\hbox { nm}$ (see “ToF BMS set-up” section). The scintillator slab is held in place by an aluminum plate with a cylindrical aperture, defining the active area of the ToF BMS to $14\hbox { mm}$ in diameter. The water-like density of the scintillator combined with its low thickness allows proton transmission at kinetic energies of $\ge 3.5\hbox { MeV}$. The emitted scintillation light is collected by an open-ended multimode fused silica fiber pointing directly at the scintillator center through a 45$^\circ$ degree drilling in the aperture plate. The high numerical aperture of 0.5 and a large core diameter of 1 mm of the fiber enable efficient light collection from the entire active scintillator area without an additional imaging system. The fiber transports the collected scintillator light onto a photodiode detector ($\textrm{PD}_{\textrm{ToF}}$ in Fig. 1a) connected to a fast oscilloscope (6 GHz; $25{\cdot }10^9\hbox { Samples/s}$). The sensitivity of the ToF BMS can be adjusted by choice of the photodiode detector. For the dose ranges $\sim 1\hbox { Gy/pulse}$ and $> 5\hbox { Gy/pulse}$, an amplified photodiode detector ($\textrm{PD}_{\textrm{ToF,1}}$, 1.5 GHz) and a non-amplified photodiode detector ($\textrm{PD}_{\textrm{ToF,2}}$, 2 GHz) are used, respectively. The advantage of this experimental setup is that the purely optical signal transport from ToF scintillator to photodiode detector/oscilloscope reduces EMP influence on the measurement as the sensitive electronic parts of the setup (photodiode and oscilloscope) can be placed in shielded housing at a distance from the EMP source. Here, increasing the length of the fiber worsens the temporal resolution of the ToF BMS signal due to dispersion effects of the polychromatic scintillation light in the fiber. A more detailed description of the components used in the ToF BMS can be found in the “ToF BMS set-up” section.

## ToF BMS workflow

### Overview

The time-resolved scintillation signal encodes the proton pulse ToF and hence the kinetic proton energy spectrum. Therefore even the raw, unprocessed signal can be used as a benchmark for LPA source stability and beamline performance. To exploit the full potential of the ToF BMS a designed workflow (Fig. 1c) for data collection and analysis has been developed. It provides calibrated proton energy spectra with real particle numbers for variable beamline settings, i.e. varying spectral and spatial pulse characteristics, through MC simulations fed with data from auxiliary measurements mainly performed within the beamline setup.

In the workflow, the first step yields relative proton spectra from the time-resolved scintillator signal by deconvolution of the detector time response function (see “Signal deconvolution” section), as well as beamline setup-specific spectral corrections (see “Signal corrections” section). The flight time reference for the protons is derived from the ToF BMS signal caused by the ultra-short laser pulse driving the proton LPA source. Since the scatterers in the beamline obstruct the optical line of sight from the LPA source to the scintillator, a second photodiode ($\textrm{PD}_{\textrm{Trig}}$ in Fig. 1a, $350\hbox { MHz}$) detecting optical emission from the LPA source is implemented and serves as reproducible starting signal for the ToF measurement. To reference the flight time of the ToF BMS signal (Fig. 1a) all scatterers are removed from the beamline (motorized for this purpose). Then the light from the ultra-short LPA laser driver is detected by the ToF BMS as it is reflected from the ToF scintillator surface and captured by the fiber (see “Flight time reference, proton energy calculation and beam divergence” section: Fig. 7a). The flight distance (distance between LPA proton source and scintillator of the ToF BMS) of $2.082\hbox { m}$, the speed of light, the starting time of the ToF BMS signal from the laser pulse and the reproducible trigger are then used to derive the proton flight time (Fig. 1$\textrm{c}_{1}$)) for the measurements with the scatterers in the beam path.

The next step in the workflow is the calibration of the relative kinetic proton energy spectrum to obtain proton numbers (Fig. 1$\mathrm {c_2}$)). The calibration is performed with the measured depth dose distribution at the irradiation site by an RCF stack. For that, the relative kinetic proton energy spectrum from the ToF BMS signal is transformed into its corresponding relative depth dose distribution (Fig. 1$\mathrm {c_3}$)) by a response matrix that includes the kinetic energy-dependent proton pulse propagation from ToF BMS to the irradiation site as well as the energy deposition in the RCF stack located there. By comparison between the measured depth dose distributions (RCF stack) and predicted relative depth dose distributions (ToF BMS spectra), a calibration factor is calculated to obtain the proton numbers at the ToF scintillator position (see “Particle number calibration of the spectrometer” section).

**Figure 2:**
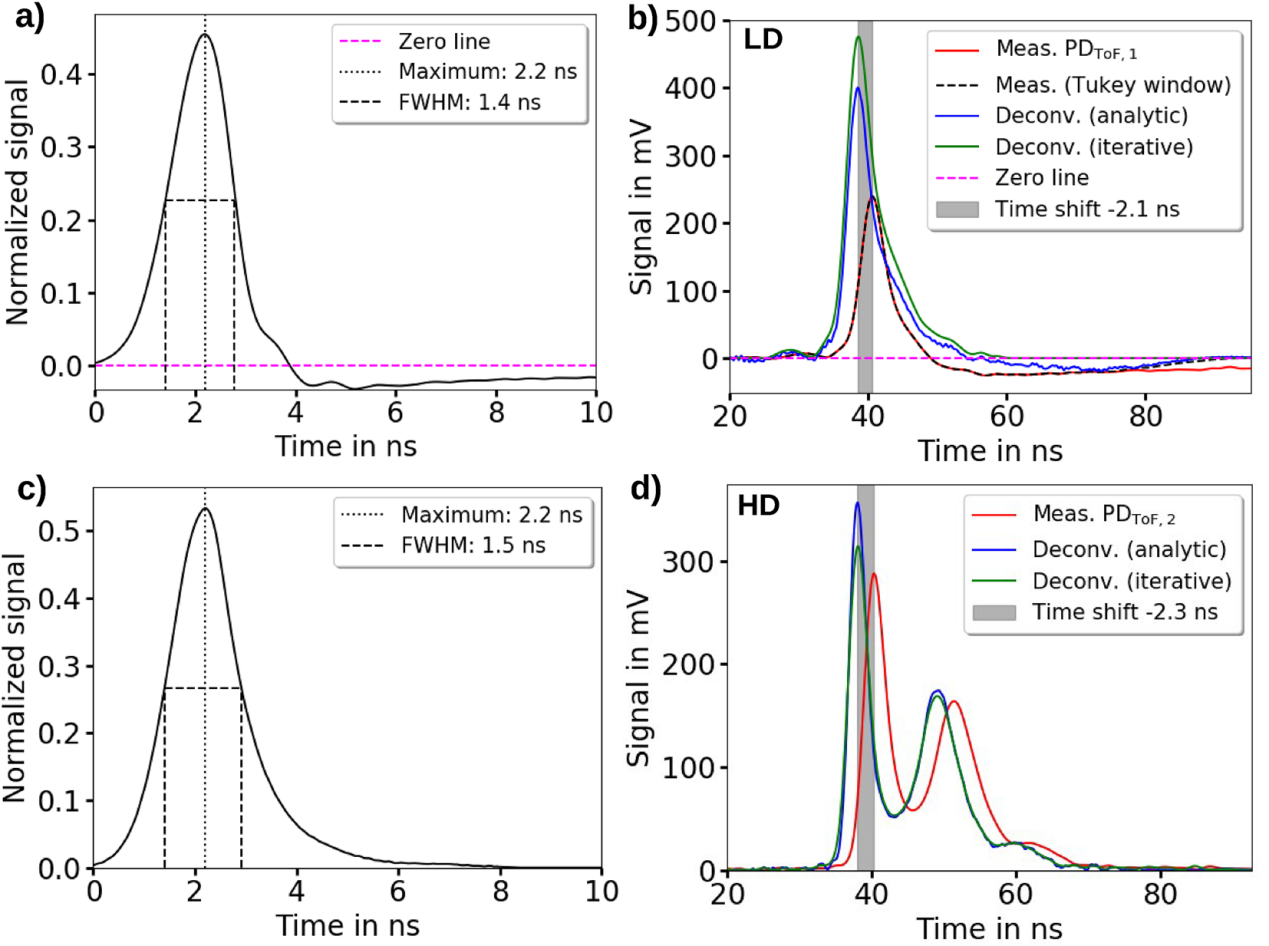
The time response functions of the time-of-flight (ToF) beam monitoring system (BMS) and the deconvolution of the measured ToF BMS signals. The measured time response functions are normalized to an area of 1 and are defined to have a time window of $2.2\hbox { ns}$ between starting point and maximum signal height, leading to a time shift of $2.2\hbox { ns}$ of the ToF BMS signal to shorter proton flight times after the deconvolution. (**a**) The ToF BMS time response function with the amplified photodiode detector $\mathrm {PD_{ToF,1}}$ used for the low dose per pulse (LD) measurements. (**b**) The analytical and iterative deconvolution of the ToF BMS signal for the LD measurements with the amplified photodiode detector $\mathrm {PD_{ToF,1}}$. Since the measured signal shows a negative ending a Tukey window is multiplied to enable the analytical deconvolution. (**c**) The ToF BMS time response function with the non-amplified photodiode detector $\mathrm {PD_{ToF,2}}$ is used for the high dose per pulse (HD) measurements. (**d**) The analytical and iterative deconvolution of the ToF BMS signal for the HD measurements.

**Figure 3:**
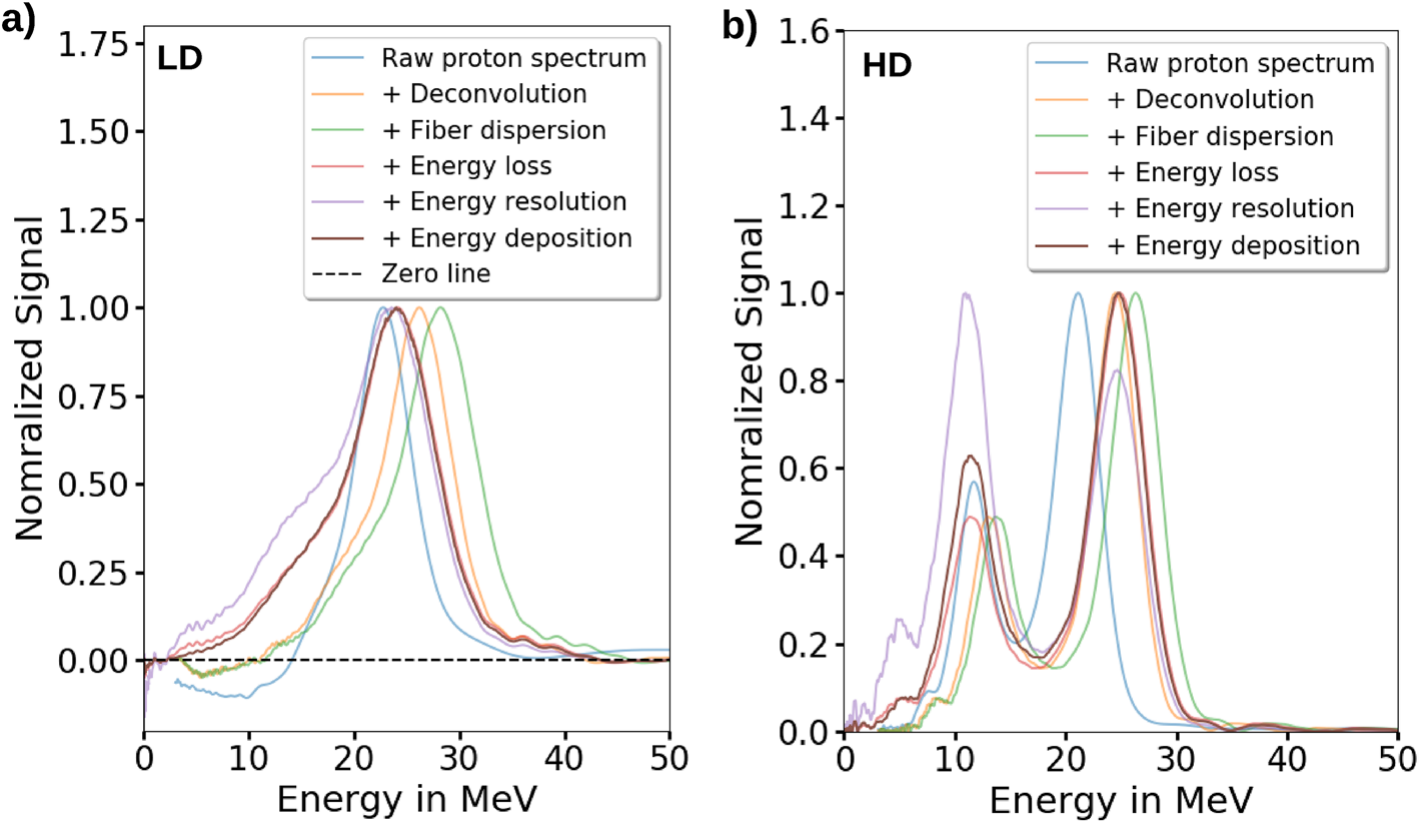
The corrections applied to the time-of-flight (ToF) beam monitoring system (BMS) signal to obtain the relative kinetic proton energy spectrum at the ToF BMS position: Deconvolution with ToF BMS time response function, dispersion of laser and scintillator light in the fiber and kinetic proton energy-dependent effects, i.e., energy loss of the protons in the scatterers, energy uncertainty of the ToF BMS and the energy deposition in the scintillator material. The step-wise spectrum corrections on the ToF BMS singal of the low dose operation mode and of the high dose operation mode are show in (**a**) and (**b**), respectively.

### Signal deconvolution

For the signal deconvolution with the time response function of the ToF BMS, the system’s response was characterized by optical excitation of the scintillator with ultra- short UV light pulses generated via a fs-laser system^40^ (see “Deconvolution of the measured ToF BMS signal” section) for both operation modes (LD/HD) and the respective photodiode detectors $\mathrm {PD_{ToF,1}}$ (Fig. 2a) and $\mathrm {PD_{ToF,2}}$ (Fig. 2c). The major difference between the response functions is the post-peak undershoot region caused by the amplifier of $\mathrm {PD_{ToF,1}}$ (Fig. 2a). The temporal resolution of the ToF BMS is $0.6\hbox { ns}$ $(1 {\upsigma })$ for both photodiode detectors, dominated by the scintillator response. Conversion of the temporal resolution into a relative energy uncertatinty of the ToF BMS follows from the setup-specific relation between flight time and kinetic proton energy which takes into account the effect of scatterers along the proton flight path (see “Flight time reference, proton energy calculation and beam divergence” section: Equation 5 and Fig. 7c). The relative energy uncertainty (see “Flight time reference, proton energy calculation and beam divergence” section: Fig. 7d) amounts to $5.5 {\%}$ $(1\upsigma )$, which is close to the system’s relative energy uncertainty of $5 {\%}$ $(1\upsigma$, for $30\hbox { MeV}$ proton energy at a flight distance of $2\hbox { m}$).

The finite rise time of the system’s response functions from signal start to maximum causes in the deconvolution a temporal shift of $2.2\hbox { ns}$ of the ToF BMS signal towards shorter flight times. The starting point of the time response function was set to the signal start at the rising edge, in order to be consistent with the signal start definition of the ToF BMS laser pulse signal (see “Flight time reference, proton energy calculation and beam divergence” section: Fig. 7a). This method is precise to a $100\hbox { ps}$-level and is confirmed by the comparison of the predicted and measured depth dose distributions. Additionally, the asymmetric shape of the time response function alters the signal shape and removes the negative part of the ToF BMS signal in the LD case (LD: Fig. 2b, HD: Fig. 2d).

For the deconvolution of the measured ToF signal, an analytical Fourier transform-based method and an iterative method adapted from Jahn et al.^41^ are used. For the analytical method, the convolution theorem is applied, i.e. the Fourier transform of the deconvoluted ToF signal is calculated by division of the Fourier transform of both the measured signal and the response function. Inverse Fourier transformation of the result then yields the deconvoluted ToF signal which is subsequently normalized by the total area (positive and negative) of the measured ToF signal.

In the iterative method, the step-wise subtraction of the response function from the ToF signal and the summation of the subtracted signals in form of symmetrical Gaussian functions centered at the corresponding beginnings of the subtracted response functions is used to calculate the deconvoluted ToF signal (see “Deconvolution of the measured ToF BMS signal” section: Fig. 6). Since the single Gaussian functions have the same area and width as the subtracted response functions, the deconvoluted signal does not require a normalization.

In Fig. 2, the performance of both deconvolution methods is compared for ToF signals of different complexity in terms of peak structure measured for LD and HD operation mode.

For the analytic deconvolution, the signal of the $\mathrm {PD_{ToF,2}}$ photodiode detector has to be multiplied with a Tukey window^42^ to remove of the post-peak undershoot. Still, the negative trail results in a reduced peak height as well as negative signal after deconvolution. Since the iterative method does not suffer from this issue, both deconvolution methods yield differences in signal height. However, since the iterative deconvolution shows that the negative part of the analytically deconvoluted ToF signal is temporally separated from the proton signal, multiplication with the Tukey window does not lead to an artifact in the analytically deconvoluted proton signal shape. The remaining signal height differences between both deconvolution methods are compensated by the calibration afterwards.

The $\mathrm {PD_{ToF,2}}$ photodiode detector (HD operation mode) does not feature an undershoot region. Instead, a peak-width dependent difference in the peak height is visible between the two deconvolution methods. As the iterative deconvolution method acts as a low-pass filter it yields a lower peak height for narrower peaks whereas broader peaks show an identical peak height for both deconvolution methods.

Owing to its property of preserving the ToF signal shape and hence information on the proton energy spectrum best, which is also confirmed by the RCF measurements (Fig. 1$\mathrm {c_3}$)), the analytic deconvolution method is applied in the further evaluation of the ToF spectra in this work.

### Signal corrections

The applied method for flight time referencing via an optical signal yields a shift of the ToF signal along the temporal axis of $1.05\hbox { ns}$. This value results from the faster travel time of the $800\hbox { nm}$ laser light (referencing the proton flight time) compared to the scintillation light at $370\hbox { nm}$ wavelength in the dispersive optical fiber of the ToF BMS setup (see “Flight time reference, proton energy calculation and beam divergence” section: Fig. 7a). For the presented ToF BMS, its integration into the ALBUS-2S beamline with scatterers along the beam path results in a more involved relation between proton flight time and kinetic energy (see “Flight time reference, proton energy calculation and beam divergence” section: Equation 5 and Fig. 7c), taking into account different proton velocities along different flight path sections. The protons’ kinetic energy loss in the scatterers as a function of the initial kinetic energy (see “Flight time reference, proton energy calculation and beam divergence” section: Fig. 7b) is obtained in a MC simulation. After this correction step the correct kinetic proton energies at the ToF BMS position can be calculated from the proton flight time values.

The nonlinear signal transformation from flight time to kinetic proton energy entails a correction of the signal amplitude, accounting for the kinetic energy-dependent sampling efficiency. Practically, each sampled amplitude is divided by the energy spread of its corresponding kinetic proton energy (see “Flight time reference, proton energy calculation and beam divergence” section: Fig. 7d).

The measured ToF signal amplitude is generated by the emission of light from the ToF scintillator, with the light emission being proportional to the energy deposited by the transversing protons. According to the energy-dependent proton stopping power yielded from MC calculations (see “Flight time reference, proton energy calculation and beam divergence” section: Fig. 7e), a correction for energy deposition is performed. After this correction step the ToF BMS signal allows to calculate the relative kinetic proton energy spectrum.

Figure 3a,b summarize the influence of the deconvolution and correction steps applied to the measured ToF BMS signal to yield the correct relative kinteic proton spectrum for the LD and HD operation mode of the ALBUS-2S beamline.

**Figure 4:**
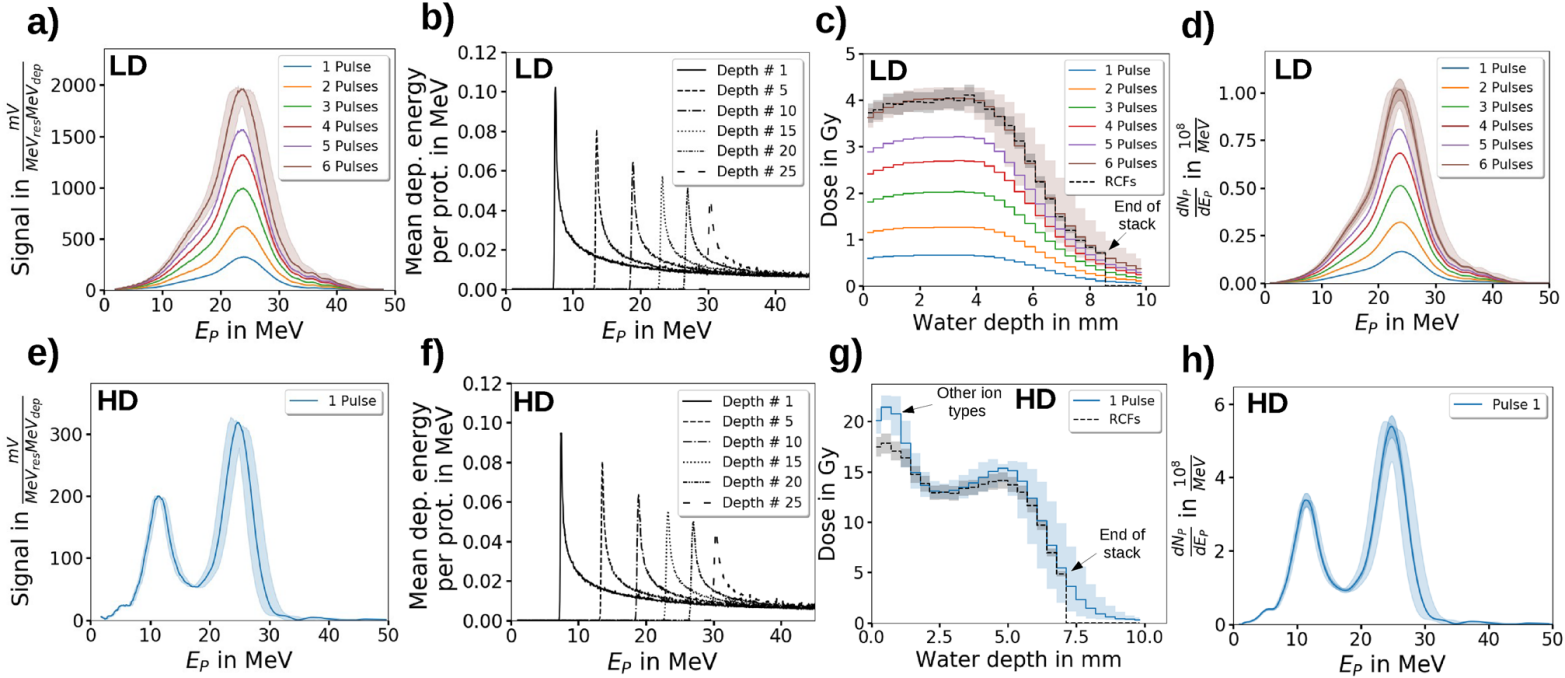
The depth dose distribution prediction from the relative kinetic proton energy spectrum for the calibration of the time-of-flight (ToF) beam monitoring system (BMS). (**a**)/(**e**) The relative kinetic proton energy spectrum measured with the ToF BMS for the low dose per pulse (LD, accumulated) / high dose per pulse (HD) operation mode. The energy uncertainty range results from the $\pm 5.5 {\%}$ ($\pm 1 {\upsigma }$) relative energy uncertainty of the ToF BMS. (**b**)/(**f**) The simulated response matrix for the proton beam propagation from the ToF BMS to the irradiation site and the energy deposition at the irradiation site for the LD/HD operation mode. It provides the kinetic proton energy -resolved mean energy deposition in the defined cylinders in the RCF stack per proton at the ToF scintillator position. The response matrix is used to transfer the measured relative kinetic proton energy spectrum to the relative depth dose distribution at the irradiation site. (**c**)/(**g**) The depth dose distribution at the irradiation site for the LD (accumulated) / HD operation mode. Normalization of the predicted relative depth dose distribution with the depth dose distribution measured by radiochromic films (RCFs) yields the calibration factor of the ToF BMS for obtaining the calibrated kinetic proton energy spectrum. The dose uncertainty range of the RCFs results from the relative dose calibration uncertainty of $\pm 5.6 {\%}$ ($\pm 2\,{\upsigma }$) of the RCFs. The dose uncertainty range of the ToF BMS predicted dose results from the relative energy uncertainty of $\pm 5.5{\%}$ ($\pm 1 {\upsigma }$) of the ToF BMS and the relative dose calibration uncertainty of $\pm 5.6{\%}$ ($\pm 2{\upsigma }$) of the RCFs. d) / g) The calibrated kinetic proton energy spectrum for the LD (accumulated) / HD operation mode. The energy uncertainty range results from the $\pm 5.5{\%}$ ($\pm 1{\upsigma }$) relative energy uncertainty of the ToF BMS and the particle number uncertainty range results from the relative dose calibration uncertainty of $\pm 5.6{\%}$ ($\pm 2{\upsigma }$) of the RCFs.

## Particle number calibration of the spectrometer

The ToF BMS shows its full potential when providing calibrated proton spectra, which is enabled through a specifically developed calibration method. The calibration is performed *in-situ*, i.e. with the complete ToF BMS integrated in the ALBUS-2S beamline as is during measurements and replaces calibration at an external proton source. Calibration can hence be performed after each change of the beamline setup - resulting in changes of transported spectra and beam envelopes - and is aligned with the inherent flexibility of the beamline setup. Moreover, changes in the device’s performance, e.g. degradation of the scintillator material or fiber, can be detected.

The *in-situ* calibration method takes the number of transported protons from the measurement of the depth dose distributions at the irradiation site using stacks of RCFs (calibrated to absorbed dose to water). To this end, the relative kinetic proton energy spectrum from the ToF BMS is converted into its corresponding relative depth dose distribution at the irradiation site via MC simulation of the beam transport from the ToF scintillator to the irradiation site (Fig. 1). In the MC simulations downstream of the ToF BMS, the proton source is defined with the lateral dimension of the ToF aperture and the relative kinetic energy distribution provided by the ToF BMS. The energy-dependent proton beam divergence (see “Flight time reference, proton energy calculation and beam divergence” section: Fig. 7f) results from the chromatic focusing properties of the beamline and the proton scattering in the scatter foils. The beam divergence is provided by dedicated RCF stack measurements at the three different positions Sc2, Ap2 and Ap3 (Fig. 1a) along the beamline. The dose deposition is simulated in the sensitive layers of a synthetic RCF stack. For this purpose, a cylindrical volume of $5\hbox { mm}$ diameter and $25{\upmu }\hbox { m}$ length (sensitive layer thickness) is defined in each layer. The dose applied to the cylindrical sensitive volumes then follows from the ratio of energy deposited by the protons and the cylinder volume and density ($1.2\hbox { g/cm}^3$ for the sensitive material^43^). The obtained cylinder dose values are then divided by the total simulated proton number to obtain the mean dose contribution per proton detected with the ToF BMS for the measured relative proton spectrum. The material’s relative water equivalent path length (WEPL) of 1.3^44^ provides the water depth of each sensitive layer. In combination with the water depth values, the simulated normalized cylinder dose values form the simulated relative depth dose distribution. For ToF BMS calibration, this is compared to the respective absorbed dose to water values from the RCF stack measurement to obtain the total proton number detected with the ToF BMS. Using a calibrated ToF BMS proton energy spectrum as the input of the MC simulation then yields the corresponding depth dose distribution at the irradiation site in absorbed dose to water values.

For a fixed beamline setup, the kinetic proton energy -dependent beam transport and energy deposition in the RCF stack can be stored in a response matrix. To obtain the depth dose distribution in the RCF stack, the response matrix is weighted with the calibrated kinetic proton energy spectrum, integrated over the kinetic proton energies and divided by the mass of the cylinder volume. This approach replaces time-consuming MC simulations and allows for a fast online depth dose distribution prediction.

The uncertainty of the RCF calibration is the main source of uncertainty for the calibration of the ToF BMS and amounts to $5.6{\%}$ for the dose levels ($4\hbox { Gy}$ accumulated/ $>12\hbox { Gy}$) applied in the LD and HD mode.

In order to predict the accumulated depth dose distribution in the irradiated RCF stack, the relative single pulse kinetic proton energy spectra obtained by the ToF BMS are accumulated (Fig. 4a) and transferred to the according depth dose distribution using the response matrix approach (Fig. 4b). Comparison of the simulated and measured depth dose distribution indicates an agreement of both distributions (Fig. 4c) and hence allows to calibrate the accumulated spectral distribution obtained by the ToF BMS (Fig. 4d). The calculated calibration factor also allows for a retrospective calibration of ToF BMS measured single-pulse spectra as well as the prediction of the single-pulse depth dose distributions in the RCF stack at the irradiation site.

In the HD operation mode, the RCF stack irradiation for the ToF BMS calibration is performed with a single proton pulse producing dose values of $>10\hbox { Gy}$. Like in the LD mode, the relative kinetic proton energy spectrum obtained by the ToF BMS (Fig. 4e) is used together with the HD mode response matrix (Fig. 4f) to predict the relative depth dose distribution in the irradiated RCF stack. Noticeable, in the HD mode, the measured and predicted depth dose distribution show a significant discrepancy in form of an overestimation of the entrance dose values in the RCF layer 1 - 4 whereas a good agreement is achieved for RCF layers > 4 (Fig. 4g). The observed differences originate from dose deposited in the ToF scintillator by other ion species produced in the acceleration process and transported by ALBUS-2S (see “Multiple ion types detected in high dose operation mode” section: Fig. 8). These ion species are stopped before reaching the irradiation site (hence not measured in the RCF stack) but are detected by the ToF BMS due to the lack of scattering foils as well as the overall higher proton energies transported in the beamline in the HD compared to the LD operation mode. Due to the discrepancy caused by other detected ion types, calculation of the total measured protons (Fig. reffig:Calibrationh) is performed by normalizing the predicted dose in the 6th RCF layer to the dose measured with the 6th RCF layer, the RCF position centered between the two dose peaks present in the HD operation mode.

**Figure 5:**
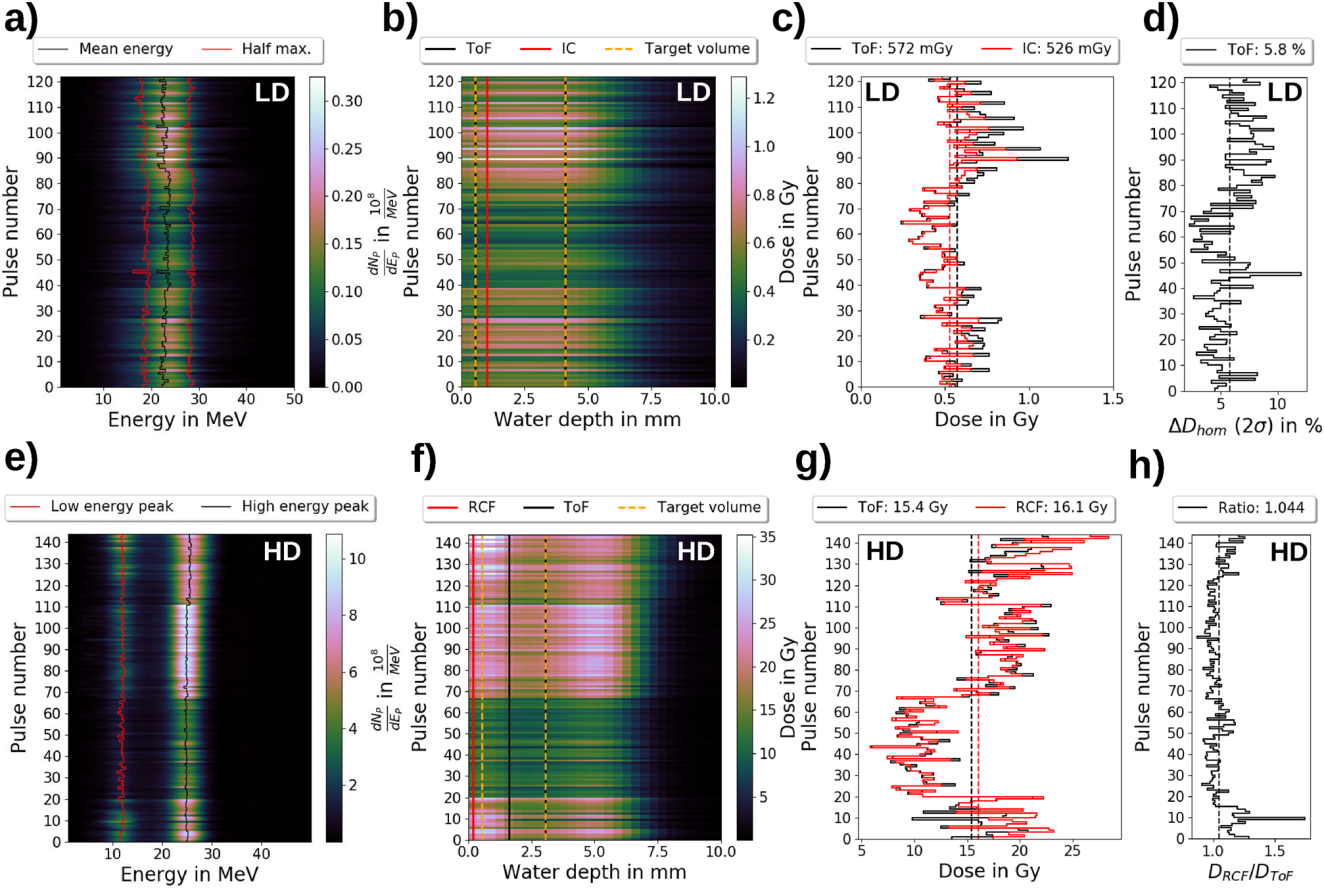
The time-of-flight (ToF) beam monitoring system (BMS) data of the transported kinetic proton energy spectra and predicted depth dose distributions at the irradiation site for the low dose per pulse (LD) and high dose per pulse (HD) operation mode. (**a**) The kinetic proton energy spectra of the LD operation mode with the mean energies and the half maximum positions. (**b**) The predicted depth dose distibutions of the LD operation in the target volume, the position of the ionization chamber (IC) dose prediction and the region for the ToF dose prediction. (**c**) The mean dose values in the ToF BMS prediction region and the dose values of the IC prediction for the LD operation mode together with the corresponding mean values. (**d**) The depth dose **inhomogeneity** values as the $2\upsigma$ standard deviations of the dose values in the ToF prediction region for the LD operation mode. (**e**) The kinetic proton energy spectra of the HD operation mode with the corresponding peak positions. (**f**) The predicted depth dose distributions of the HD operation mode with the target volume, the position of the radiochromic film (RCF) dose measurement and the region for the ToF dose prediction. (**g**) The mean dose values in the ToF prediction region and the measured dose values by the single RCFs for the HD operation mode together with the corresponding mean values. (**h**) The depth dose inhomogeneity as the ratios of the measured RCF dose values and the mean dose values in the ToF prediction region.

## Beam monitoring of laser plasma-based accelerated proton pulses

The evaluation of ToF BMS spectra and corresponding MC-simulated depth dose distibutions of $>100\hbox { pulses}$ for both LD and HD operation mode of the ALBUS-2S beamline showcase the device’s capabilities for LPA source stability analysis as well as prediction of application-specific figures of merit for dose distributions.

### Low dose operation mode

As described previously, the LD operation mode of the ALBUS-2S beamline uses pulse accumulation to mitigate LPA-inherent source fluctuations and targets a dose of 4 Gy, which is required to be applied with a deviation of $\le \pm 10{\%}$ and a depth dose inhomogeneity of $\le 10{\%}$ in a target volume of 5 mm diameter and 4 mm length. Here, the ToF BMS is applied to monitor 122 single pulse kinetic proton energy spectra (Fig. 5a). The transported spectra feature a high spectral stability with an average mean kinetic proton energy and FWHM of $23\hbox { MeV}\pm 3 {\%}$ ($\pm 1{{\upsigma }_{\textrm{stat}}}$) and $10\hbox { MeV}\pm 5{\%}$ ($\pm 1{{\upsigma }_{\textrm{stat}}}$), respectively, whereas the number of protons per pulse reflects the LPA source fluctuations ($1.8{\cdot }10^8{}\pm 56{\%}$ ($\pm 2{{\upsigma }_{\textrm{stat}}}$)). Here, $\mathrm {{\upsigma }_{stat}}$ is the statistical standard deviation and for the paticle number the $2{{\upsigma }_{\textrm{stat}}}$-standard deviation is evaluated since the fluctuations in the particle number directly translate into fluctuations of the dose value applied to the target volume.

To evaluate if the achieved stability in terms of pulse spectrum and intensity complies with the operation mode’s requirements, the respective predicted depth dose distributions (Fig. 5b) and derived figures of merit such as the single pulse mean dose values (Fig. 5c) and the depth dose inhomogeneity (Fig. 5d) in the target volume are dereived. The ToF BMS predicts an average mean pulse dose of $572\hbox { mGy}\pm 58{\%}$ ($\pm 2{{\upsigma }_{\textrm{stat}}}$), ensuring a $95 {\%}$-probability of a single pulse dose between $\sim 240\hbox { mGy/pulse}$ and $\sim 900\hbox { mGy/pulse}$. This range slightly exceeds the desired range from $330\hbox { mGy/pulse}$ to $800\hbox { mGy/pulse}$. With the measured total proton pulse length of $20\hbox { ns}$, a single pulse dose rate in the order of $10^8\hbox { Gy/s}$ is achieved. The ToF BMS predicted dose values are verified by comparison with dose values from a calibrated ionization chamber (IC) operated in transmission downstream from the ToF BMS (Fig. 1a) with an effective measurement location at $1.06\hbox { mm}$ water depth in the target volume (Fig. 5b). The calibration of the transmission IC is performed with a calibrated advanced Markus IC located at the irradiation site. Owing to the high dose rate of the LPA proton pulses, the measured IC dose values at the irradiation site have to be corrected for saturation effects (charge recombination), which is performed with the model of Gotz et al.^45^ by using a correction factor between 1.05 to 1.35 for measured pulse dose values between 200 mGy and 900 mGy. The mean dose values in the target region predicted by ToF BMS and measured with the transmission IC are generally in good agreement, yet for pulse dose values $>\sim 600\hbox { mGy}$, the transmission IC measurements are lower than the ToF BMS predictions. This observation can indicate remaining uncorrected saturation effects in the advanced Markus IC placed at the irradiation site for cross-calibraton of the transmission IC as the shape agreement of depth dose distributions predicted by the ToF BMS and measured with the dose rate independent RCF stacks renders dose nonlinearities in the ToF measurement unlikely.

The predicted depth dose inhomogeneity is $5.8 {\%}$ ($2\upsigma$), which translates into a maximal dose difference between maximum and minimum depth dose value in the target region of $11.6 {\%}$ relative to the applied mean dose value in the target region. This value slightly exceeds the desired $\le 10{\%}$ inhomogeneity but pulse accumulation has shown to reduce the depth dose inhomogeneity^31^.

### High dose operation mode

In the HD operation mode of the ALBUS-2S beamline, a mean single pulse dose of $>10\hbox { Gy}$ is required together with a depth dose inhomogeneity of $\le 10 {\%}$ in the target volume of 5 mm diameter and 3 mm length. Here, the ToF BMS is used to characterize 144 single pulse kinetic proton energy spectra (Fig. 5e). The transported kinetic proton energy spectra obtained by the ToF BMS feature a low kinetic energy peak at $12\hbox { MeV}\pm 3 {\%}$ ($\pm 1 {{\upsigma }_{\textrm{stat}}}$) with a peak height (proton number) of $2.4{\cdot }10^8{1/\hbox {MeV}}\pm 89 {\%}$ ($\pm 2{{\upsigma }_{\textrm{stat}}}$) and a high kinetic energy peak at $25\hbox { MeV}\pm 3{\%}$ ($\pm 1{{\upsigma }_{\textrm{stat}}}$) with a peak height of $6.7{\cdot }10^8{1/\hbox {MeV}}\pm 63{\%}$ ($\pm 2 {{\upsigma }_{\textrm{stat}}}$). The stability of the peak energies is determined by the beamline setting whereas fluctuations of the peak heights originate from LPA source instabilities. As discussed above, in the HD operation mode, other ion types co-propagating with the low energy peak protons are transported to the ToF BMS (see “Multiple ion types detected in high dose operation mode” section: Fig. 8), contributing to the observed lower peak height stability compared to the high energy peak. For the corresponding MC-based depth dose profile predictions (Fig. 5f), the contribution from other ion species at the ToF BMS position causes an overestimation of the target volume’s entrance dose (Fig. 4g). Hence, the second half of the target volume (from $1.6\hbox { mm}$ to $3.1\hbox { mm}$ water depth) is used for dose predictions and compared to dose values measured by a single RCF layer placed in front of the target volume (Fig. 5f). The predicted mean dose (ToF BMS, Fig. 5g) amounts to $15.4\hbox { Gy}\pm 57{\%}$ ($\pm 2{{\upsigma }_{\textrm{stat}}}$), with minimum and maximum doses of $6.9\hbox { Gy}$ and $22.9\hbox { Gy}$, respectively. With a measured total proton pulse length of $20\hbox { ns}$, a single pulse dose rate in the order of $10^9\hbox { Gy/s}$ is calculated. The average depth dose inhomogeneity in the target volume is estimated as the ratio between the entrance dose (RCF measurement) and the volume dose in the second half of the target volume (ToF BMS prediction) and amounts to 1.044 (Fig. 5h). This value translates into an average maximum dose difference of $4.4 {\%}$ between entrance volume dose and the volume dose in the second half of the target volume, which is well in agreement with the required $\le 10 {\%}$ inhomogeneity for the HD beamline operation mode. Moreover, the high level of correlation for RCF and ToF BMS dose values suggests that the ToF BMS can be applied for dose predicitons even in the multi-$10\hbox { Gy/pulse}$ range at ultra-high dose rates where conventional ICs suffer from severe saturation. In the presented data set, six different groups in terms of achieved dose can be identified (pulse 1 to 5, 6 to 15, 16 to 20, 21 to 67, 68 to 111, 112 to 144), corresponding to six different experimental runs on several days. On day 1 to 6, average volume dose values of $17.1\hbox { Gy}$, $15.0\hbox { Gy}$, $15.3\hbox { Gy}$, $10.2\hbox { Gy}$, $18.7\hbox { Gy}$ and $18.0\hbox { Gy}$, respectively, are predicted. As the beamline ALBUS-2S was operated the same on all six days, the differences in achived dose originate from the LPA source performance, determining the available number of protons in the beamline’s angular and spectral acceptance range. Ultimately, the ToF BMS is a sensitive tool to investigate the influence of daily laser setup variations on LPA source performance, with the goal to maximize performance in terms of achievable dose at the irradiation site.

## Conclusion

In this work, we have presented a first time-of-flight-based (ToF-based) spectrally resolving beam monitor operated online and in transmission for single laser plasma-based accelerator (LPA) proton pulse characterization at beamline setups. The device is accompanied by a specifially developed workflow providing calibrated proton spectra with a relative energy uncertainty of $5.5 {\%}$ $(1 {\upsigma })$. The current relative energy uncertainty of the system is dominated by the scintillator materials temporal resolution and could be reduced to the level of $1.9 {\%}$ $(1{\upsigma }$, considering fiber dispersion and photodiode detector time response) without increasing the flight distance by implementation of a faster/quenched scintillator as e.g. BC-422Q with $5{\%}$ (weight) of benzophenone and a temporal resolution of $94\hbox { ps}$ $(1{\upsigma })$.

The low relative energy uncertainty forms the basis for Monte-Carlo simulation-based predictions of the depth dose distribution. By making both spectral and dose distribution-related information accessible, the ToF beam monitoring system (BMS) provides unique data regarding LPA proton source fluctuations as well as their effect on application-relevant parameters for corresponding dose distributions as e.g. applied in radiobiological studies. Ultimately, this approach enables the feasibility assessment for potential experiments at LPA beamlines based on predicted dose distributions without performing the generally time-consuming characterization of volumetric dose distributions.

Besides partly substituting radiochromic film stack -based characterization of depth dose distributions, the ToF BMS can also provide dose predictions in measurement regimes characterized by high dose and ultra-high dose rate where ionization chambers (ICs) face saturation isssues.

Specificially for the ALBUS-2S beamlines - but other tunable beamline concepts as well - the ToF BMS can be used to characterize the LPA proton source spectrum by actively selecting different transported proton energy bands. This capability can be exploited for fast online feedback on the LPA process and beamline performance by generating input data for automated feedback loops for LPA source tuning e.g. aided by novel data handling methods such as machine learning.

In summary, we believe that theses unique features in combination with the electromagnetic pulse hardness and the option for *in-situ* calibration of the device will make the optical ToF BMS an attractive transmission beam monitoring device for LPA proton beamlines, comparable to transmission ICs for conventional proton accelerators.

## Methods

### ToF BMS set-up

The used scintillator in the ToF BMS is the BC-422Q scintillator from Saint-Gobain. It is a plastic scintillator quenched with $0.5{\%}$ (weight) of benzophenone in order to reach a response time of $153\hbox { ps}$ $(1{\upsigma })$. The faster timing comes at the expense of total light output. The fact that the measured temporal resolution of the ToF BMS yielded $0.6\hbox { ns}$ $(1 {\upsigma })$ indicates that the benzophenone might have evaporated from the only $200{\upmu }\hbox { m}$ thick scintillator layer over a timescale of multiple years. This assumption can be confirmed by using the detector time response $FWHM_{PD,ToF,1}=0.4\hbox { ns}$, the time dispersion of the scintillator light ($360\hbox { nm}-420\hbox { nm}$, BC-422) in the fiber $FWHM_{Fiber}=0.36\hbox { ns}$ and the scintillator temporal resolution $FWHM_{Scint}=1.3\hbox { ns}$ (BC-422). Those values leading to a temporal resolution of the ToF BMS of

1 $$\begin{aligned} {\Delta }t_{ToF} = \frac{\sqrt{FWHM_{Scint}^2+FWHM_{Fiber}^2+FWHM_{PD,ToF,1}^2}}{2\sqrt{2ln(2)}}\approx \frac{1.4\hbox { ns}}{2\sqrt{2ln(2)}}\approx 0.6\hbox { ns}\quad (1\upsigma ) \end{aligned}$$

which fits with the measured response function and the calculated temporal resolution of the ToF BMS.

For transporting the emitted scintillator light, a $15\hbox { m}$-long fused silica multi-mode fiber (FP1000URT, Thorlabs) with a wavelength range of 300 nm to 1200 nm is used.

$\mathrm {PD_{ToF,1}}$ is a modified version of the amplified photodiode detector FDP 310-FC-VIS ($1.5\hbox { GHz}$, MenloSystems). The original photodiode of this detector was replaced with the S5973-01 photodiode (Hamamatsu), which has a ball lens for focusing the light transported by the fiber onto the sensitive silicon chip, leading to a strong signal increase. Additionally the built-in amplifier increases the output voltage signal strength by $+20\hbox { dB}$. $\mathrm {PD_{ToF,2}}$ is a SV2-FC photodiode detector ($2\hbox { GHz}$, Thorlabs) with the originally installed S5973-01 photodiode (Hamamatsu). $\mathrm {PD_{Trig}}$ is a DET10A photodiode detector (Thorlabs) with a $1\hbox { ns}$ risetime.

A $25{\cdot }10^9\hbox { Samples/s}$, 6GHz Oscilloscope (Tektronix MSO64) is used to sample the voltage signal from the photodiode detectors.

### Deconvolution of the measured ToF BMS signal

The response function is determined by optical excitation of the scintillator at a wavelength of 257.5 nm, i.e. below its emission wavelength of 370 nm. The ultra-short laser pulses with 260 fs pulse duration are generated as the 4th harmonic of a $\mathrm {Yb^{3+}:CaF_2}$ laser system which provides laser pulses of 160 fs in the millijoule range at a wavelength of 1030 nm^40^. To reproduce the ToF BMS setup as closely as possible during the response function measurement, the fiber ending collecting the scintillator light emission was placed under a $45{^\circ }$ angle. A glass plate (BK7) in front of the fiber which is transparent to the scintillator emission but absorbs the UV laser light acted as a wavelength separator of excitation and scintillation photons. The obtained response function of the ToF BMS is used to deconvolute the measured ToF signal. For the deconvolution of the measured ToF signal, an analytical Fourier transform-based method and an iterative method are used.

**Figure 6:**
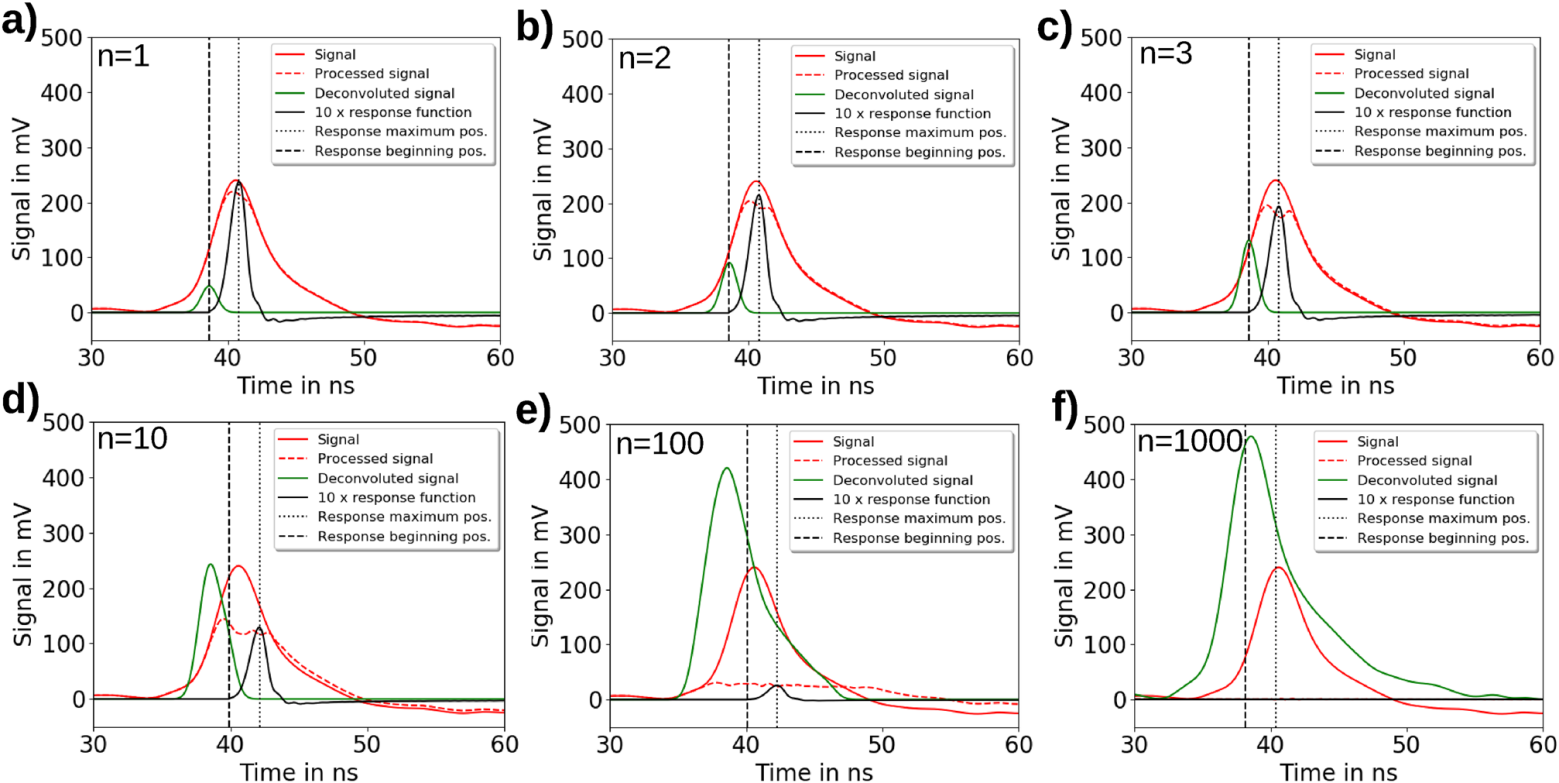
The iterative algorithm used for deconvolution of the measured time-of-flight (ToF) signal with the ToF beam monitoring system (BMS) time response function. Here, the red solid line is the measured ToF signal and the red dashed line is the processed ToF signal resulting from the iterative subtraction of the ToF BMS time response function shown as the black solid line. The green solid line shows the resulting deconvoluted ToF BMS signal. At the beginning, the processed ToF signal is identical with the measured ToF signal. During the iterative deconvolution process the ToF BMS response function maximum (dotted black line) gets aligned for best overlap with the processed ToF signal and the response function maximum is normalized to $10 {\%}$ of the processed ToF signal hight at this position. Then the ToF BMS response is subtracted from the processed ToF signal and then added to the deconvoluted signal as a symmetrical Gaussian function with the same area and FWHM, centered at the ToF BMS response function beginning (dashed black line). This process is repeated until the processed ToF signal reaches a stable minimum. The iteration steps 1, 2, 3, 10, 100 and 1000 are shown in (**a**)–(**f**), respectively.

In the iterative deconvolution method (Fig. 6) is based on a five step iteration process:

1. Maximum of ToF BMS response function is normalized to maximum of measured ToF signal
2. Convolution between ToF BMS response function and measured ToF signal is used to align ToF system response function on the flight time axis for best overlap with ToF signal.
3. Maximum of ToF BMS response function is normalized to $10{\%}$ of the signal height of the measured ToF signal at maximum position of ToF BMS response function on the flight time axis.
4. ToF BMS response function is subtracted from the measured ToF signal.
5. Gaussian function with the area and FWHM of the subtracted ToF BMS response function centered at the beginning of ToF BMS response function on the flight time axis is added to the deconvoluted ToF signal.

Since the area under the deconvoluted signal is already identical with the area of the measured ToF signal no further normalization of the deconvoluted ToF signal is required.

### Flight time reference, proton energy calculation and beam divergence

**Figure 7:**
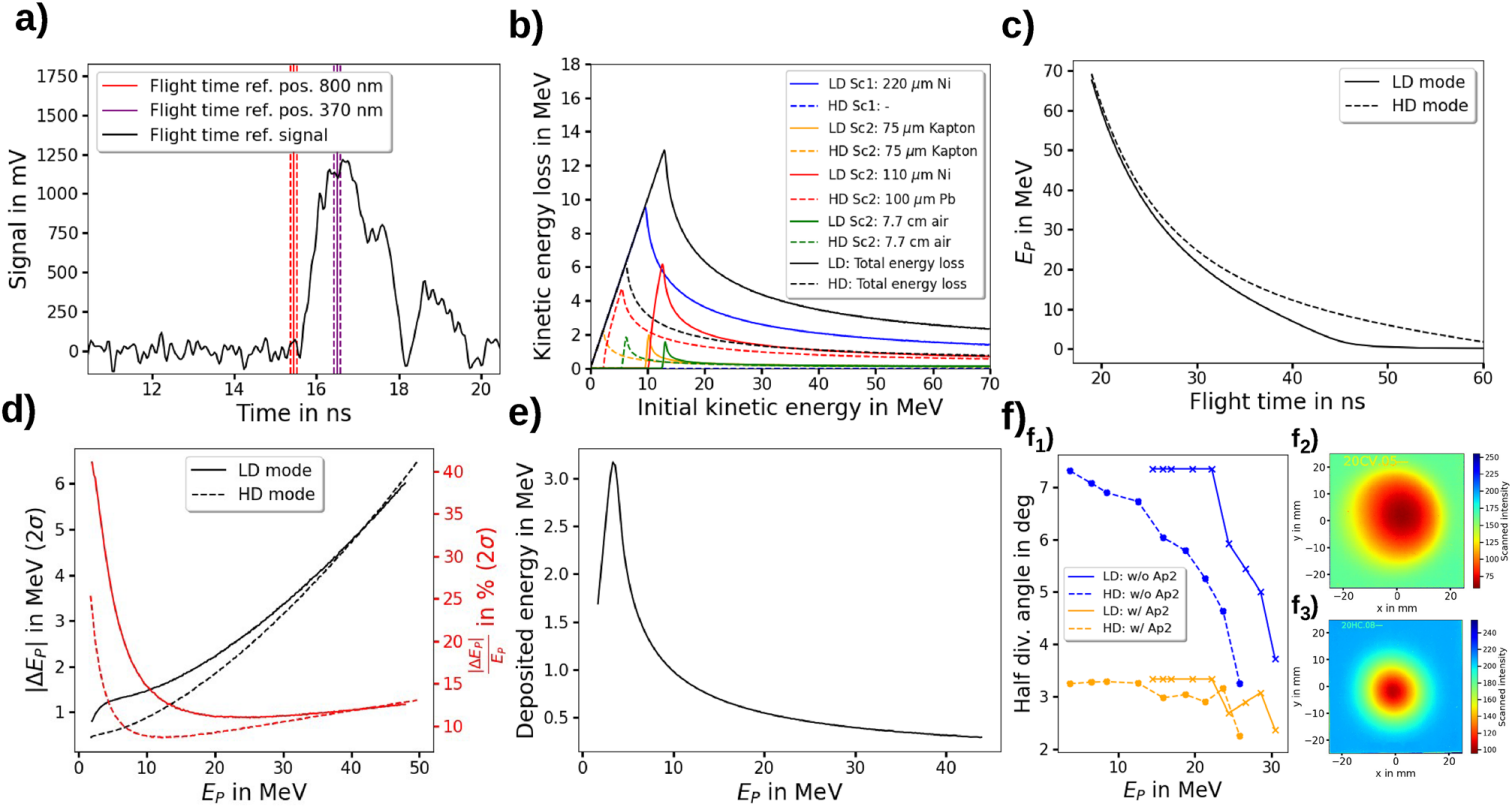
The required relations for the calculation of the proton flight time, kinetic energy spectrum and beam propagation for the two beamline operation modes (low dose per pulse (LD) and high dose per pulse (HD)). (**a**) The measured proton flight time reference produced by the 800 nm laser light and the calculated 370nm proton flight time reference considering the dispersion in the fiber. The uncertainty of $\pm 75\hbox { ps}$ originates from the rise time of the potodiode detector of 150 ps. (**b**) The energy loss of the protons in the scatterers, the Kapton window and the air distance depending on their initial kinetic energy. (**c**) The simulated relation between the flight time and the kinetic proton energy at the position of the time-of-flight (ToF) beam monitoring system (BMS). (**d**) The simulated kinetic proton energy uncertainty of the ToF BMS dependent on the proton energy at ToF BMS position. (**e**) The deposited energy in the scintillator dependent on the proton energy at ToF BMS position. (**f**) The proton beam divergence and profile. $\mathrm {f_1)}$ The energy-resolved proton beam divergence at ToF BMS position with and without ToF aperture for LD and HD operation mode. $\mathrm {f_2)}$ The proton beam profile (red channel of scanned RCF image) at Ap3 position for the LD operation and proton energies $\ge 22.2\hbox { MeV}$. $\mathrm {f_3)}$ The proton beam profile (green channel of scanned RCF image) at Ap2 position for the HD operation and proton energies $\ge 23.7\hbox { MeV}$.

To reference the proton flight time, the beginning of the ToF BMS laser signal $t_{laser}$ (Fig. 7a) is measured separately without the scattering foils in the beamline. Here, $t_{laser}$ is cross-referenced to the proton ToF BMS signals measured with the scattering foils in place via a reference photodiode detector ($\mathrm {PD_{Trig}}$) detecting the light of the laser plasma interaction and triggering the oscilloscope. Since the beginning of this signal is induced by 800 nm laser light, one has to consider the dispersion ${\Delta }n_f=0.021$ between the 800 nm laser light and the 370 nm scintillator light in the fused silica fiber with a length $l_f=15\hbox { m}$. The corresponding time shift ${\Delta }t_n$ is given by

2 $$\begin{aligned} {\Delta }t_n =\frac{{\Delta }n_fl_f}{c} = 1.05\hbox { ns}. \end{aligned}$$

Here $c\approx 3{\cdot }10^8{\mathrm {m/s}}$ is the speed of light. Furthermore, one has to consider the travel time of the laser light $t_{ToF,l}$ from the LPA proton source to the scintillator plate ($d_{ToF}=2.082\hbox { m}$), which is given by

3 $$\begin{aligned} t_{ToF,l}=\frac{d_{ToF}}{c}=6.94\hbox { ns}. \end{aligned}$$

The resulting proton flight time value $t_{ToF,p}$ is then given by

4 $$\begin{aligned} t_{ToF,p} = t_{trig,p}-t_{laser}-{\Delta }t_n+t_{ToF,l}. \end{aligned}$$

Here, $t_{trig,p}$ is the time given by the oscilloscope referenced by the triggering event.

The energy resolved energy loss of the protons during their flight to the ToF BMS in the scattering foils, Kapton window and air is quantified by MC simulations and shown for the LD and HD operation mode in Fig. 7b. In the LD operation mode a $220 {{\upmu }\hbox { m}}$ thick Nickel foil was located at the distance $d_{Sc1}=182\hbox { cm}$ to the proton source causing a kinetic proton energy loss of $E_{p,Sc1}^{loss}$ and a $75 {\upmu }\hbox { m}$ thick Kapton window, a $110{\upmu }\hbox { m}$ thick Nickel foil and a $7.7\hbox { cm}$ long air distance located at $d_{Sc2}=200.5\hbox { cm}$ causing a kinetic proton energy loss of $E_{p,Sc2}^{loss}$. For the HD operation mode the scatter foil at $d_{Sc1}$ was removed and the $110{\upmu }\hbox { m}$ thick Nickel foil at $d_{Sc2}$ was replaced by a $100{\upmu }\hbox { m}$ lead foil.

The flight time information of the protons $t_{ToF,p}$ is then used together with the MC-simulated kinetic energy loss values $E_{p,Sc1}^{loss}$ and $E_{p,Sc2}^{loss}$ in Equation 5 to calculate the kinetic proton energy values $E_{ToF,p}$ at the ToF BMS position. The resulting relation between proton flight time $t_{ToF,p}$ and kinetic proton energy at the ToF scintillator $E_{ToF,p}$ for the LD and HD operation mode is plotted in Fig. 7c.

5 $$\begin{aligned} t_{ToF,p}=\frac{1}{c}\left( \frac{d_{Sc1}}{\sqrt{1-\frac{1}{\left( \frac{E_{ToF,p}+E_{p,Sc1}^{loss}+E_{p,Sc2}^{loss}}{E_{p,rest}}+1\right) ^2}}}+\right. \left. \frac{d_{Sc2}-d_{Sc1}}{\sqrt{1-\frac{1}{\left( \frac{E_{ToF,p}+E_{p,Sc2}^{loss}}{E_{p,rest}}+1\right) ^2}}}+\frac{d_{ToF}-d_{Sc2}}{\sqrt{1-\frac{1}{\left( \frac{E_{ToF,p}}{E_{p,rest}}+1\right) ^2}}}\right) . \end{aligned}$$

The energy uncertainty of the ToF BMS for the for the LD and HD operation mode is shown in Fig. 7d. In Fig. 7e the MC-simulated kinetic proton energy- resolved energy deposition in the scintillator plate is shown.

To simulate the response matrices required for the prediction of the depth dose distribution the kinetic proton energy dependent beam divergence was measured with three RCF stacks at the three different positions Sc2, Ap2 and Ap3 (Fig. 1a) and implemented in the MC simulations. The kinetic energy resolved proton beam divergence for the LD and HD operation mode is shown in Fig. 7f.

### Multiple ion types detected in high dose operation mode

**Figure 8:**
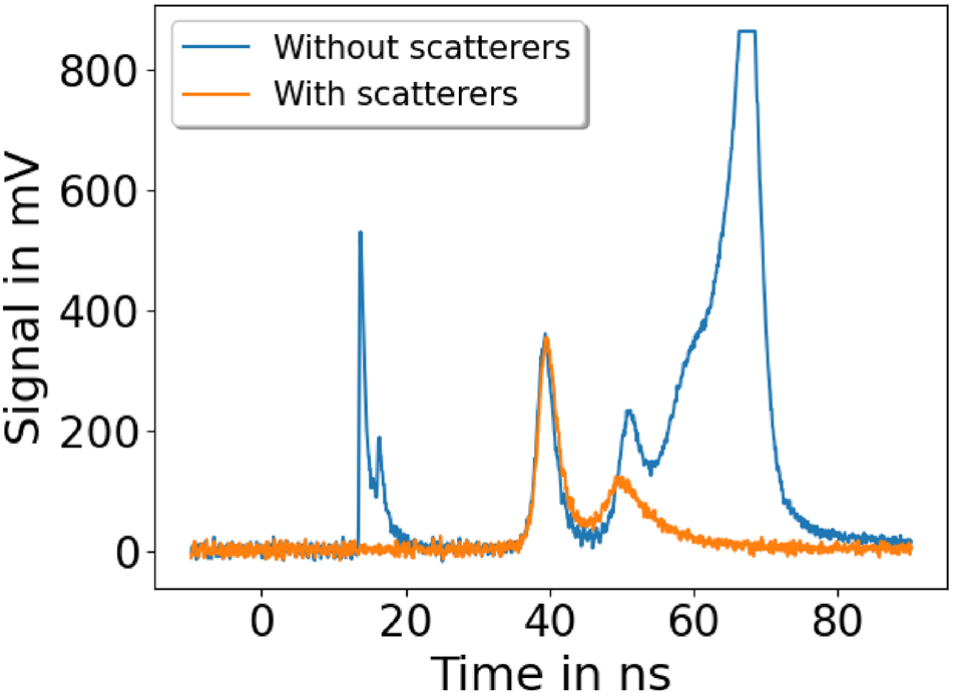
The time-of-flight beam monitoring system signal during high dose operation mode with and without $100 {\upmu }\hbox { m}$ lead scatter foil at Sc2 position. Without scatter foil, the laser light induced zero signal at $\sim 15\hbox { ns}$ used for flight time reference and transported ion types other than protons are visible. The other ion types are producing the strong signal (clipped due to limited dynamic range) after the two proton peaks at $\sim 40\hbox { ns}$ and $\sim 50\hbox { ns}$. The second proton peak is temporally overlapping with the other ion types.

In the HD operation mode of the ALBUS-2S beamline a discrepancy between the predicted depth dose distribution with the ToF BMS signal and the measured depth dose distribution with the RCF stack is observed. This effect can be explained with ion types other than protons that are transported through the solenoids along with lower energetic protons. Using the $100{\upmu }\hbox { m}$ lead scatter foil at the Sc2 position, one can strongly suppress ion types other than protons (Fig. 8), but cannot prevent that the fastest ions or their fragments reach the ToF scintillator and produce scintillation light, which is detected by the ToF BMS. Since ions heavier than protons are too low in energy to reach the irradiation site, their contribution to the measured ToF BMS signal is not related to the dose measured by the RCF stack at the irradiation site. This explains why the predicted entrance dose by the meaured ToF BMS signal is too high compared to the measured dose with the RCF stack.

### FLUKA

The Monte Carlo (MC) simulations in this work were performed with FLUKA (version 4-0.0). It is a fully integrated particle physics MC simulation package developed at CERN^46^. The simulations were performed with the standard settings of FLUKA for hadron therapy applications using the “HADROTHErapy” card.

### RCF

For the dose measurements radiochromic EBT3 films from the company GAFchromic were used. They consist of an active layer protected by two polyester layers. The thickness and atomic composition of the two different layer types required for the implementation in the FLUKA MC simulation are shown in Table 1. The RCFs are calibrated for absorbed dose to water with proton beams at the University Proton Therapy Dresden facility at the center of a spread out Bragg peak (SOBP) produced with an initial proton energy of $150\hbox { MeV}$ using a dedicated double-scattering field formation device. The relative WEPL of the RCFs was found to be 1.3^44^. To obtain the dose values, the irradiated RCFs are scanned and the increase of the optical density in the red channel ($\le 12\hbox { Gy}$) or in the green channel ($>12\hbox { Gy}$) of the obtained rgb image is evaluated. The relative calibration uncertainty of the red and green channel is $5.6 {\%}$ ($2\upsigma$). The RCFs are used to measure the equivalent 3D dose distribution in water at the irradiation site, by irradiating them in a stack configuration consisting of multiple films.

**Table 1:** Atomic composition of EBT3 film layers from the company GAFchromic^43^.

Region	Density	Thickness	H	Li	C	O	Al
	in $\mathrm {g/cm^3}$	in $\mathrm {{\upmu }m}$			in $\mathrm {\%}$		
Polyester layer	1.35	125	36.4	–	45,5	18.2	–
Active layer	1.2	25	56.8	0.6	27.6	13.3	1.6

### ICs

For the dose monitoring, a transmission IC (type 7862, PTW) is used, which is cross-calibrated to dose values measured at the irradiation site using an advanced Markus chamber (AMC, type 34045, PTW). The air-filled open AMC is calibrated for absorbed dose to water by $^{60}$Co photon irradiation. The chamber readout is corrected for the proton radiation quality, air density and dose-rate dependent saturation with the charge recombination model for plane parallel ICs from Gotz et al.^45^.

## Data Availability

The datasets generated and/or analysed during the current study are available in the Data publication: Time-of-Flight spectroscopy for laser-driven proton beam monitoring repository, https://rodare.hzdr.de/record/1832.
